# Performability evaluation, validation and optimization for the steam generation system of a coal-fired thermal power plant

**DOI:** 10.1016/j.mex.2022.101852

**Published:** 2022-09-10

**Authors:** Subhash Malik, Shubham Verma, Arun Gupta, Gaurav Sharma, Shakuntla Singla

**Affiliations:** a Department of Mechanical Engineering, MMEC, Maharishi Markandeshwar (Deemed to be) University, Mullana, India; b Department of Methamatics, MMEC, Maharishi Markandeshwar (Deemed to be) University, Mullana, India; c SKIET, Kurukshetra, India

**Keywords:** Markov method, Mnemonic rule, Normalizing condition, Performability model, Artificial neural network, Particle swarm optimization

## Abstract

The present paper talks over performability evaluation for a steam generation system of a Coal Fired Thermal Power Plant (CFTPP) using the concept of the Markov method. A steam generation system provides a suitable amount of steam for the sound functioning of the plant. The system comprises five subsystems, i.e., High-Pressure Heater, Economizer, Boiler Drum, Water Tubes, and Super Heater.  First, the transition diagram of the concerned system is designed based on the state probabilities of various subsystems. The differential equations are derived based on the mnemonic rule. After that, the performability model is developed by using the normalizing condition. The performability levels for various subsystems are obtained by placing the appropriate value of failure and repair rates in the developed model. The performability of each subsystem is evaluated based on performability matrices. It is observed that the economizer subsystem is most critical in which the availability increased from 0.7640 to 0.8827, i.e. (11.87 %). In contrast, boiler drum is the least crucial subsystem with availability enhanced from 0.8627 to 0.8657 (i.e., 0.3 %). The results show that the economizer subsystem must be given top priority, and the boiler drum be given the least priority from the maintenance outlook. The performability levels obtained through the Markov method are compared with those obtained through the Artificial Neural Network to validate. Moreover, machine learning (artificial neural network) and optimization technique (particle swarm optimization) is also employed to check the adequacy of the results and optimized process parameters.

• The aim of the present study is evaluate the performance of steam generation system of a coal fired thermal power plant.
• The probabilistic approach (i.e. Makov Method) is used to formulate the transition diagram of the steam generation system. Then, the first-order differential equations are obtained using the mnemonic rule and further solved recursively.
• The results show that the economizer system must be given top priority, and the boiler drum subsystem must be given the least priority from the maintenance outlook.

## Graphical abstract

**Figure:**
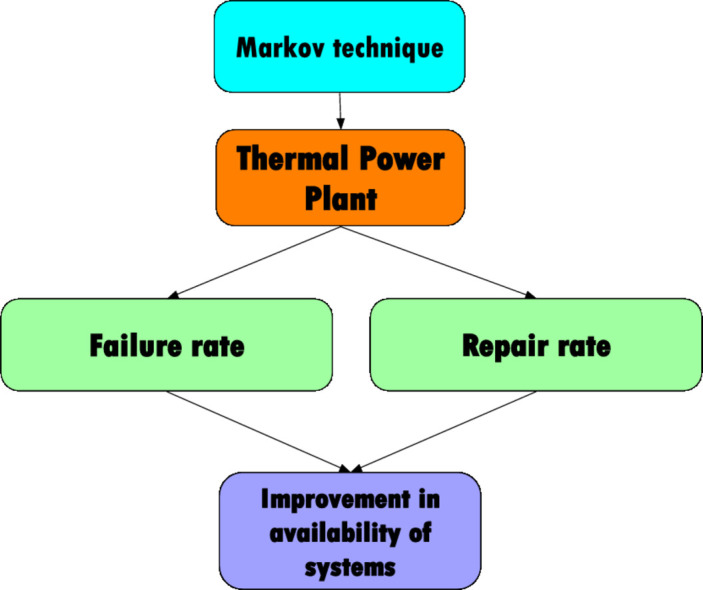

Specification table

**Table:** 

Subject area: More specific subject area: Method name Name and reference of original method	Engineering Realiability and Maintainability Markov Process Statiscal approach for improvement in the maintainability and reliability of the system. Braglia, M., Carmignani, G., Frosolini, M. and Zammori, F. (2012), “Data classification and MTBF prediction with a multivariate analysis approach”, Reliability Engineering and System Safety, Vol. 97, No. 1, pp. 27–35.
Resource availability	Thermal power plant in India

## Materials method

Steam is used to produce electricity by spinning a turbine, which is connected to a generator. It is generated in the thermal power plant at high pressure by the combustion of coal within the boiler. In this competitive world, every industrialist wants that their plants run without failure to get the highest profit. Therefore, it is essential that every part/equipment ought to run without breakdown and provide magnificent performance. The failure/breakdown is an inevitable case, and various parts/equipment are subjected to random failure [1]. Reliability engineering is focused on the systematic study of failures of engineering subsystems/systems in a specified working environment for a given time and their remedies [2]. This discipline gives the industry numerous essential tools and concepts to enhance its performability through effectively utilizing input resources [3]. Many researchers have examined steady-state availability, reliability, and maintenance analysis of multifaceted process industries using different numerical methods. Further, many more techniques were used to improve the performance of various industrial systems. Ansari et al. [4] proposed a mathematical model to find the maximum power from the photovoltaic panel system with the help of the PSO technique. This optimization technique was compared with Perturb and Observe (PO) at various insulations and temperatures. PSO technique was found more suitable to obtain the maximum power from the photovoltaic panel system. Bahl et al. [5] gave a simulation modeling to find out the behavior of the distillery plant by considering the different input parameters for various components and determining the highest critical components, which greatly influenced the availability of the plant. Braglia et al. [6] discussed an oil refinery plant with a multivariate statistical approach to support the classification of mechanical components working in a specific environment. An effective preventive maintenance plan was formulated by assessing the impact of working conditions on the mean time between failures. Chokshi et al. [7] evaluated the thermal performance of each plant's equipment at different loads and the effect of critical parameters on them. Artificial Neural Networking (ANN) is used to estimate the energetic and exergetic performance of the plant. Deep and Bansal [8] used the PSO algorithm to obtain optimum machining parameters of the CNC Lathe machine to reduce the machining time and improve the machining quality. Fiondella et al. [9] presented a statistical method to determine the production system's reliability parameters with the help of a discrete-time Markov approach. Garg et al. [10] described the mathematical model to evaluate the reliability and maintenance schedule for the block-board manufacturing system in the plywood industry. Gupta et al. [11] calculated the availability of a generator in a steam turbine power plant using the exponential distribution with the help of the Markov Birth-Death process. Gupta et al. [12] described a method to compute availability, mean time before failure, and reliability for a butter manufacturing plant. The ordinary differential equations have been solved by using the fourth-order Runga-Kutta method to obtain the reliability of the butter manufacturing plant. Kaur et al. [13] discussed a numerical method to obtain the transient state for systems having inconsistent failure and repair rates. Kumar et al. [14] analyzed the availability of a system in a thermal power plant with the help of the Markov approach and suggested the maintenance schedule for various subsystems of the system concerned. Marinakis et al. [15] proposed the Ant Colony (ACO and the PSO algorithms to solve the financial classification model. They tested the proposed methods through two different financial classification problems. Yazdi [16] discussed a heuristic optimization model using fuzzy numbers and triangular intuitionistic to find the reliability of the model and used Spearman Correlation. Liu et al. [17] discussed the maintenance strategies of the product under warranty period having limited repair time and repair number. Further, they discussed the repair cost of the product is based on the repair time. Rajpal et al. [18] described the use of artificial neural networking (ANN) to determine a repairable system's performance. The neural network was trained with the help of past plant data. The simulation results were used to formulate a strategy for the optimum working of the system. Chen [19] discussed the preemptive scheduling with a single machine to minimize the total weighted late work and weighted number of tardy jobs. Further, they also discussed the Pareto-scheduling problem. Kumari et al. [20] discussed the solution of constrained problems using particle swarm optimization (PSO). Li and Zhang [21] used dynamic programming to solve the redundancy allocation optimization for the multi-state series and parallel systems. Zeng and Sun [22] analyzed the competing failure in the system based on stochastic Petri nets in their study and also discussed the effect of common cause failure of the system. Moreover, Table 1 shows the comparison of previous published work.

**Table 1:** Comparison of previous published research work.

Name	Year	Method/ Technique	Plant
Adhikary et al. [1]	2012	RAM Investigation of Coal-Fired Thermal Power Plants by using Markov Method	210 MW coal-fired thermal power plant in eastern region of India.
Aggarwal et al. [2]	2015	Markov modeling and reliability analysis of urea synthesis system	Urea synthesis system of a fertilizer plant
Arora and Kumar [3]	1997	Availability analysis of steam and power generation systems	thermal power plant
Ansari et al. [4]		Particle swarm optimization technique	PV System
Bahl et al. [5]	2018	Availability analysis of distillery plant using Petri Nets	Distillery plant
Braglia et al. [6]	2012	multivariate statistical approach	an oil refinery plant
Chokshi et al. [7]	2018	Artificial Neural Network	thermal power plants
Gupta et al. [11]	2020	Markov Method	steam turbine powerplants

In the current scenarios, automation in industries is a vital problem owing to massive capital investment. For example, a power plant industry requires a multifaceted system with tremendous capital investment and planning. Besides this, the failure of components is also another problem related to this industry. To overcome all these problems, a proper arrangement for preventive maintenance must be necessary. Thus, a mannered maintained schedule is required for all the components of the power plant industry. In addition, the power plant industry equipment's are always working in harsh conditions. Because of this, it requires to repair and replacement of components from time to time. It means the condition of the machine depends upon the operating conditions. These conditions may be different for different systems. The priority for the power plant industry is to retain the availability of all the systems during the process. The availability of the plant can be measured on the basis of operational time without failure. In current research work, the main focus is to preserve the plant in working condition without any failure by maintaining the different systems (i.e., steam generation) of a plant in a failure-free State. It is a vital part of the power plant industry and consists of five subsystems organized in sequences. The outcome of the concerned system is interconnected with the reliability and maintainability of the equipment. It depends upon the number of failures. For this, an optimal maintenance strategy is required by taking the highest maintenance priority to the most critical subsystem of the system. In this current research, a birth-death Markov method is utilized for solving the above-mentioned problems related to power plant industries. The transition diagram for different power plant industry systems is drawn as per the working condition of the system. Moreover, the differential equations are generated for each system by utilizing the transition diagram. In addition, the performance model is designed as per the transition diagram. Finally, performance analysis of various subsystems is measured as per the decision matrix obtained from the developed performance model.

## Steam generation system description

**Fig. 1:**
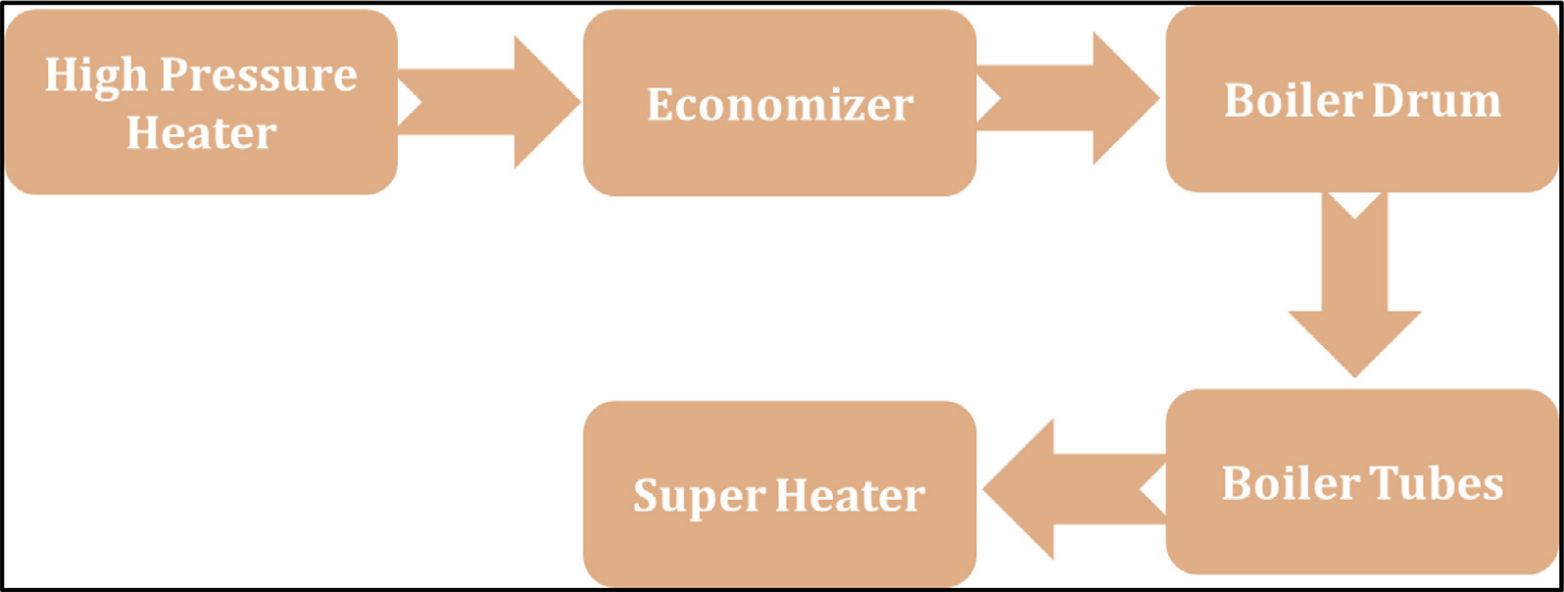
Schematic flow diagram for steam generation system.

A steam generation system ensures a regular supply of steam for the efficient execution of a thermal power plant. The concerned system comprises mainly five subsystems (as shown in Figure 1) in a chain configuration with the following description:

(i) High-Pressure Heater (HP): It has three high-pressure heaters in series. The function of a high-pressure heater is to increase the temperature of feed water. If one of them fails, the system goes into a complete failure state.
(ii) Economizer (EC): It is used to capture the heat from the flue gases and transfer it to the boiler feed water.  It consists of a single economizer; its failure causes the complete failure of the concerned operating system.
(iii) Boiler Drum (BD): It comprises one boiler drum to separate the saturated steam from the steam-water mixture. Its failure causes the whole failure of the system.
(iv) Boiler Tubes (BT): Hot gasses come from the furnace and contact with water tubes where the heat of these hot gases transfers to the water, and consequently, steam is produced in the boiler. Failure of the water tube causes complete failure of the system.
(v) Super Heater (SH): It is a device that converts saturated steam into superheated steam. Superheater failure leads to the whole shutdown of the system.

**Notations**

H, E, D, T and S : Show the operating state of the subsystems of HP, EC, BD BT and SH respectively. h, e, d, t and s : Indicate that failed state of the subsystems HP,EC,BD, BT and SH

P_S0_ ($\hat{t}$) : Probability of the system working with full capacity at time t.

P_S1_($\hat{t}$)-P_S5_($\hat{t}$) : Probabilities of the system in a failed state.

F_i,_ i=1-5 : Mean failure rates of HP, EC, BD, BT and SH respectively.

R_i,_ i=1-5 : Mean repair rates of HP, EC, BD, BT and SH respectively.

K_i_: It is ration of failure rate (F_i_) to repair rate (R_i_)

(') : Represents first order derivative w.r.t time (t).

: Denotes the working of the system without failure.

: Exhibits the reduced capacity of the system.

: Signifies the system in failed condition.

**Assumptions**

(i) Initially, all the subsystems are working in full.
(ii) Zero waiting time for repair facilities.
(iii) The performance of a repaired component performs like a new one for a particular time period.
(iv) The exponential distribution will be followed by failure and repair rates.
(v) The subsystems cannot fail simultaneously.

**Fig. 2:**
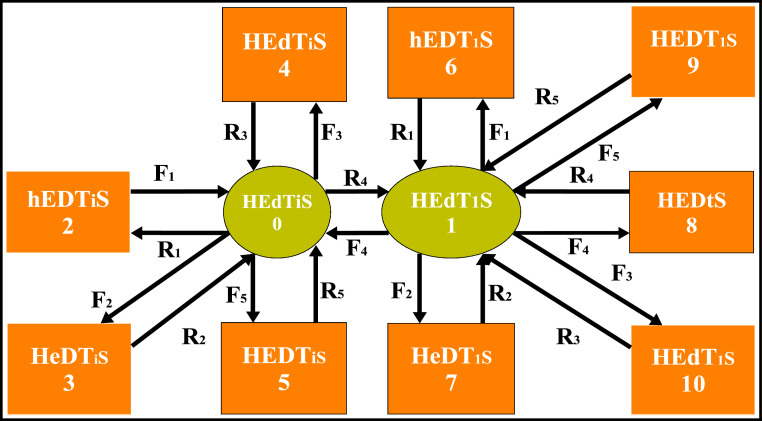
Transition diagram for steam generation system.

The transition diagram for the steam generation system consists of 11 states as depicted in Figure 2. The initial state (S0) is working with full capacity. State S1 is a reduced capacity state, and S2 to S10 shows that the system is in a failed state due to the complete failure of one or the other subsystem of the steam generation system.

## Mathematical formulation using Chapman-Kolmogrove differential equations

The differential equations associated with the transition diagram derived by using the mnemonic rule are as follows:

(3.1) $$\begin{array}{ccc} & & P_{S0}^{\prime }\left(\hat{t}\right)+F_{1}P_{S0}\left(\hat{t}\right)+F_{2}P_{S0}\left(\hat{t}\right)+F_{3}P_{S0}\left(\hat{t}\right)+F_{4}P_{S0}\left(\hat{t}\right)+F_{5}P_{S0}\left(\hat{t}\right)\;\\ & & =R_{4}P_{S1}\left(\hat{t}\right)+R_{1}P_{S2}\left(\hat{t}\right)+R_{2}P_{S3}\left(\hat{t}\right)+R_{3}P_{3S4}\left(\hat{t}\right)+R_{5}P_{S5}\left(\hat{t}\right) \end{array}$$

(3.2) $$\begin{array}{ccc} & & {P^{\prime }}_{S1}\left(\hat{t}\right)+R_{4}P_{S1}\left(\hat{t}\right)+F_{1}P_{S1}\left(\hat{t}\right)+F_{2}P_{S1}\left(\hat{t}\right)+F_{3}P_{S1}\left(\hat{t}\right)+F_{4}P_{S1}\left(\hat{t}\right)+F_{5}P_{S1}\left(\hat{t}\right)\\ & & =\;F_{4}P_{S0}\left(\hat{t}\right)+\;R_{4}P_{S8}\left(\hat{t}\right)+R_{1}P_{S6}\left(\hat{t}\right)+R_{2}P_{S7}\left(\hat{t}\right)+R_{3}P_{3S10}\left(\hat{t}\right)+R_{5}P_{S94}\left(\hat{t}\right) \end{array}$$

(3.3) $$P_{S2}^{\prime }\left(\hat{t}\right)+R_{1}P_{S2}\left(\hat{t}\right)=F_{1}P_{S0}\left(\hat{t}\right)$$

(3.4) $$P_{S3}^{\prime }\left(\hat{t}\right)+R_{2}P_{S3}\left(\hat{t}\right)=F_{2}P_{S0}\left(\hat{t}\right)$$

(3.5) $$P_{S4}^{\prime }\left(\hat{t}\right)+R_{3}P_{S4}\left(\hat{t}\right)=F_{3}P_{S0}\left(\hat{t}\right)$$

(3.6) $$P_{S5}^{\prime }\left(\hat{t}\right)+R_{5}P_{S5}\left(\hat{t}\right)=F_{5}P_{S0}\left(\hat{t}\right)$$

(3.7) $$P_{S6}^{\prime }\left(\hat{t}\right)+R_{1}P_{S6}\left(\hat{t}\right)=F_{1}P_{S1}\left(\hat{t}\right)$$

(3.8) $$P_{S7}^{\prime }\left(\hat{t}\right)+R_{2}P_{S7}\left(\hat{t}\right)=F_{2}P_{S1}\left(\hat{t}\right)$$

(3.9) $$P_{S8}^{\prime }\left(\hat{t}\right)+R_{4}P_{S8}\left(\hat{t}\right)=F_{4}P_{S1}\left(\hat{t}\right)$$

(3.10) $$P_{S9}^{\prime }\left(\hat{t}\right)+R_{5}P_{S9}\left(\hat{t}\right)=F_{5}P_{S1}\left(\hat{t}\right)$$

(3.11) $$P_{S10}^{\prime }\left(\hat{t}\right)+R_{3}P_{S10}\left(\hat{t}\right)=F_{3}P_{S1}\left(\hat{t}\right)$$

With initial conditions at the time $\hat{t}$ = 0

Ps_i_ ($\hat{t}$) = 1 for i=0,

Ps_i_ ($\hat{t}$) = 0 for i ≠ 0

**Solution of equations by steady state behavior**

The steady-state or long-run behavior of the system can be analyzed by setting P**^'^** = 0 as t→∞. The equations from (3.1) to (3.11) are written as:

(3.12) $$\begin{array}{ccc} & & F_{1}P_{s0}\left(\hat{t}\right)+F_{2}P_{S0}\left(\hat{t}\right)+F_{3}P_{S0}\left(\hat{t}\right)+F_{4}P_{S0}\left(\hat{t}\right)+F_{5}P_{S0}\left(\hat{t}\right)\;\\ & & =R_{4}P_{S1}\left(\hat{t}\right)+R_{1}P_{S2}\left(\hat{t}\right)+R_{2}P_{S3}\left(\hat{t}\right)+R_{3}P_{S4}\left(\hat{t}\right)+R_{5}P_{S5}\left(\hat{t}\right) \end{array}$$

(3.13) $$\begin{array}{ccc} & & R_{4}P_{S1}\left(\hat{t}\right)+F_{1}P_{S1}\left(\hat{t}\right)+F_{2}P_{S1}\left(\hat{t}\right)+F_{3}P_{S1}\left(\hat{t}\right)+F_{4}P_{S1}\left(\hat{t}\right)+F_{5}P_{S1}\left(\hat{t}\right)\\ & & =\;F_{4}P_{S0}\left(\hat{t}\right)+\;R_{4}P_{S8}\left(\hat{t}\right)+R_{1}P_{S6}\left(\hat{t}\right)+R_{2}P_{S7}\left(\hat{t}\right)+R_{3}P_{S10}\left(\hat{t}\right)+R_{5}P_{S9}\left(\hat{t}\right) \end{array}$$

(3.14) $$R_{1}P_{S2}\left(\hat{t}\right)=F_{1}P_{S0}\left(\hat{t}\right)$$

(3.15) $$R_{2}P_{S3}\left(\hat{t}\right)=F_{2}P_{S0}\left(\hat{t}\right)$$

(3.16) $$R_{3}P_{S4}\left(\hat{t}\right)=F_{3}P_{S0}\left(\hat{t}\right)$$

(3.17) $$R_{5}P_{S5}\left(\hat{t}\right)=F_{5}P_{S0}\left(\hat{t}\right)$$

(3.18) $$R_{1}P_{S6}\left(\hat{t}\right)=F_{1}P_{S1}\left(\hat{t}\right)$$

(3.19) $$R_{2}P_{S7}\left(\hat{t}\right)=F_{2}P_{S1}\left(\hat{t}\right)$$

(3.20) $$R_{4}P_{S8}\left(\hat{t}\right)=F_{4}P_{S1}\left(\hat{t}\right)$$

(3.21) $$R_{5}P_{S9}\left(\hat{t}\right)=F_{5}P_{S1}\left(\hat{t}\right)$$

(3.22) $$R_{3}P_{S10}\left(\hat{t}\right)=F_{3}P_{S1}\left(\hat{t}\right)$$

**Solving these Eqs. (12)–(22) recursively,**

$P_{S1}=K_{4}P_{S0}$, $P_{S2}=K_{1}P_{S0}$, $P_{S3}=K_{2}P_{S0}$

$P_{S4}=K_{3}P_{S0}$, $P_{S5}=K_{5}P_{S0}$$P_{S6}=K_{1}\;K_{4}P_{S0}$

$P_{S7}=K_{2}K_{4}P_{S0}$, $P_{S8}=K_{4}K_{4}P_{S0}P_{S9}=K_{4}K_{5}P_{S0}$$P_{S10}=K_{3}K_{4}P_{S0}$

$K_{i}=\;F_{i}/R_{i}$ i = 1, 2, 3, 4, 5

Use of normalizing condition i.e., the sum of all the state probabilities is equal to one [$\sum_{i=0}^{10}P_{\text{Si}}=1$], gives the solution as follows:

$$P_{S0}+P_{S1}+P_{S2}+P_{S3}+P_{S4}+P_{S5}+P_{S6}+P_{S7}+P_{S8}+P_{S9}+P_{S10}=1$$

By putting the value of different stages ($P_{S1}to\;P_{S10}$) in the form of $P_{S0}$

$$P_{S0}=1/\;\left[1+\left(1+K_{4}\right)\left(K_{1}+K_{2}+K_{3}+K_{4}+K_{5}\right)\right]$$

The performability model of the steam generation system is obtained by the addition of reduced and full working state probabilities.

$$\text{Performability}\;\text{Model}=P_{S0}+P_{S1}$$

(23) $$\text{Performability}\;\text{Model}=\left(1+K_{4}\right)P_{S0}$$

## Performability evaluation

**Fig. 3:**
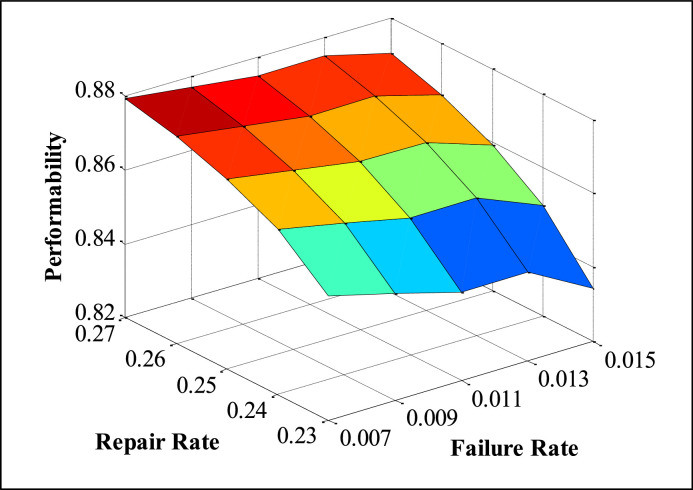
Effect of FRR of HPH on system performability.

**Fig. 4:**
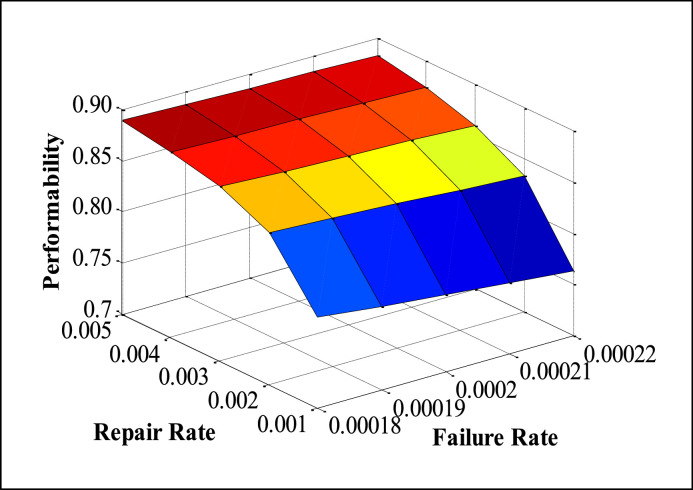
Effect of FRR of economizer on system performability.

**Fig. 5:**
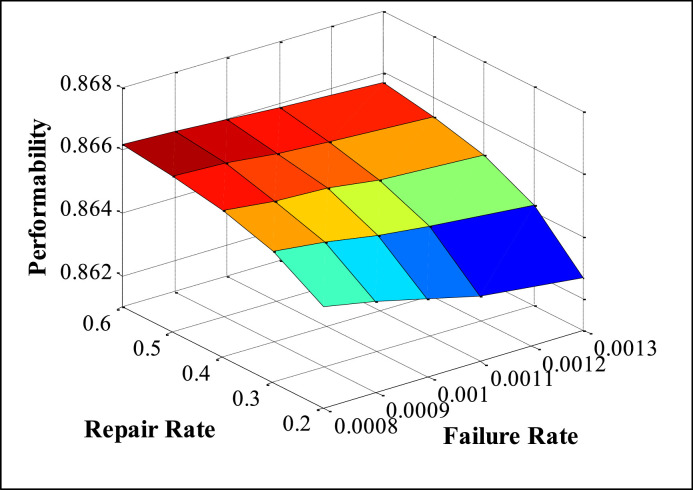
Effect of FRR of boiler drum on system performability.

**Fig. 6:**
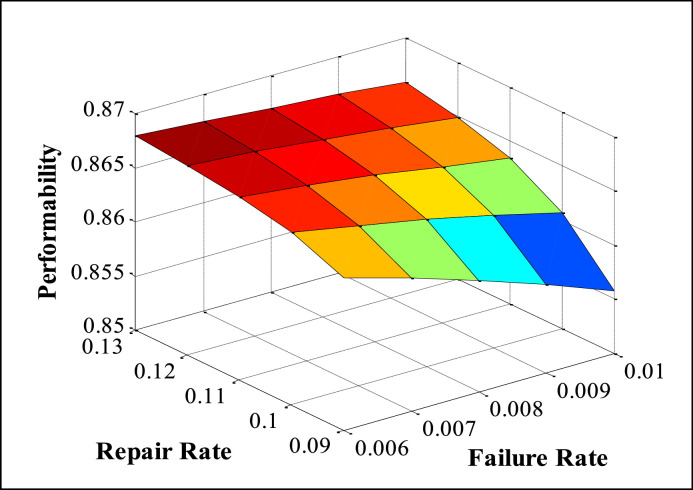
Effect of FRR of boiler tubes on system performability.

**Fig. 7:**
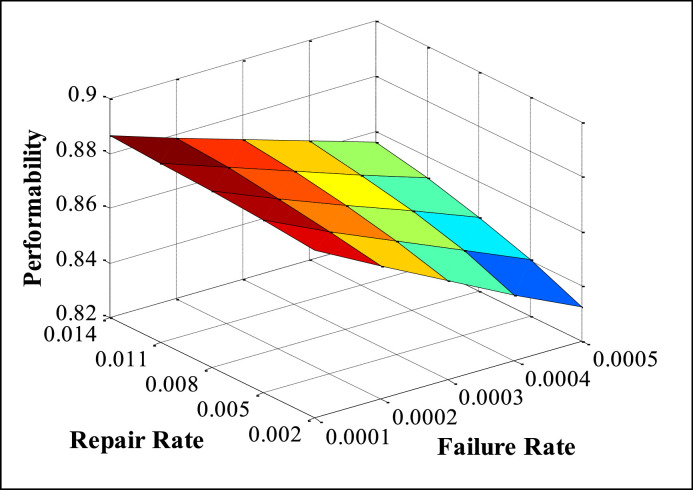
Effect of FRR of superheater on system performability.

The appropriate values of FRR (Failure and Repair Rate) are taken from maintenance records available in the history cards, maintenance sheets, etc., and also on the basis of discussion with concerned plant employees. The performability of subsystems is obtained by placing the suitable values of FRR in the developed model and solved in MATLAB. The numerous performability levels of different subsystems have been presented in Table 2, Table 3, Table 4, Table 5, Table 6, as well as in Fig. 3, Fig. 4, Fig. 5, Fig. 6, Fig. 7, respectively. The impact of FRR on various subsystems of steam generation system is shown below:

**Table 2:** Performability matrix for HPH of steam generation system.

R_1_, F_1_	0.23	0.24	0.25	0.26	0.27	Constant Parameters
**0.007**	0.8535	0.8646	0.8714	0.8760	0.8793	**F_2_=0.00020 R_2_=0.003** **F_3_=0.0010 R _3_=0.4** **F_4_=0.008 R_4_=0.11** **F_5_=0.0003 R_5_=0.008**
**0.009**	0.8487	0.8609	0.8684	0.8734	0.8771	
**0.011**	0.8439	0.8572	0.8654	0.8709	0.8749	
**0.013**	0.8392	0.8535	0.8624	0.8684	0.8727	
**0.015**	0.8345	0.8499	0.8594	0.8659	0.8705

**Table 3:** Performability matrix for economizer of steam generation system.

R_2_, F_2_	0.001	0.002	0.003	0.004	0.005	Constant Parameters
**0.00018**	0.7881	0.8482	0.8704	0.8819	0.8889	**F_1_=0.011 R_1_=0.25** **F_3_=0.0010 R _3_=0.4** **F_4_=0.008 R_4_=0.11** **F_5_=0.0003 R_5_=0.008**
**0.00019**	0.7819	0.8446	0.8679	0.8799	0.8874	
**0.00020**	0.7758	0.8411	0.8654	0.878	0.8858	
**0.00021**	0.7699	0.8376	0.8629	0.8761	0.8842	
**0.00022**	0.7640	0.8341	0.8604	0.8742	0.8827

**Table 4:** Performability matrix for boiler drum of steam generation system.

R_3_, F_3_	0.2	0.3	0.4	0.5	0.6	Constant Parameters
**0.0008**	0.8642	0.8652	0.8657	0.8660	0.8662	**F_1_=0.011 R_1_=0.25** **F_2_=0.0020 R _3_=0.003** **F_4_=0.008 R_4_=0.11** **F_5_=0.0003 R_5_=0.008**
**0.0009**	0.8639	0.8650	0.8655	0.8659	0.8661	
**0.0010**	0.8635	0.8647	0.8654	0.8657	0.8660	
**0.0011**	0.8631	0.8645	0.8652	0.8656	0.8659	
**0.0012**	0.8627	0.8642	0.8650	0.8654	0.8657

**Table 5:** Performability matrix for boiler tubes of steam generation system.

R_4_ F_4_	0.09	0.10	0.11	0.12	0.13	Constant Parameters
**0.006**	0.8640	0.8659	0.8669	0.8675	0.8679	**F_1_=0.011 R_1_=0.25** **F_2_=0.00020 R _3_=0.003** **F_3_=0.0010 R _3_=0.4** **F_5_=0.0003 R_5_=0.008**
**0.007**	0.8622	0.8648	0.8662	0.8670	0.8675	
**0.008**	0.8603	0.8636	0.8654	0.8664	0.8670	
**0.009**	0.8581	0.8622	0.8644	0.8657	0.8665	
**0.010**	0.8558	0.8607	0.8634	0.8649	0.8659

**Table 6:** Performability matrix for superheater of steam generation system.

R_5_F_5_	0.002	0.005	0.008	0.011	0.014	Constant Parameters
**0.0001**	0.8812	0.8831	0.8845	0.8856	0.8864	**F_1_=0.011 R_1_=0.25** **F_2_=0.0021 R _3_=0.003** **F_3_=0.001 R _3_=0.4** **F_4_=0.008 R_4_=0.11**
**0.0002**	0.8685	0.8721	0.8748	0.8769	0.8787	
**0.0003**	0.8561	0.8614	0.8654	0.8685	0.8710	
**0.0004**	0.8440	0.8509	0.8561	0.8602	0.8635	
**0.0005**	0.8323	0.8407	0.8470	0.8520	0.8561

Table 2 presents the performability analysis of the steam generation system. The maximum value of performability, i.e., 0.8705, is obtained at a failure rate of 0.015 and repair rate of 0.27. Fig. 3 depicts the 3D surface plot between repair rate and failure rate for HPH. It is observed that the performability is enhanced up to 3.60 % when the failure and repair rate varies between 0.007 to 0.015 and 0.23 to 0.27, respectively.

The variation in performability of the Economizer subsystem is shown in Table 3. It is concluded that the maximum performability is observed at a failure rate of 0.00022 and a repair rate of 0.005. Fig. 4 illustrates the graphical analysis of the performability of for Economizer subsystem. The failure rate (F_2_) of the Economizer is increased from 0.00018 to 0.00022, thus resulting in a decrement in the performability from 0.7881 to 0.7640. Conversely, results are obtained for the repair rate.

The performability analysis for the boiler drum subsystem is depicted in Table 4 and Fig. 5. The system performability is enhanced from 0.8627 to 0.8657 with a failure rate, and the repair rate varies between 0.0008 to 0.0012 and 0.2 to 0.6, respectively.

Table 5 and Fig. 6 reveal the performability analysis of the boiler tube subsystem. The failure rate (F_4_) of Boiler Tubes increases from 0.006 to 0.010, and the performability of the system decreases merely from 0.8640 to 0.8558 i.e., 0.82%. In the same way, as the repair rate (R_4_) increases from 0.09 to 0.13, the performability of the system increases just from 0.8558 to 0.8659 i.e., 1.01%.

Table 6 and Fig. 7 indicate the effect of FRR of the superheater subsystem on the performability of the steam generation system. When the failure rate (F_5_) of the superheater increases from 0.0001 to 0.0005, then the performability of the system decreases noticeably from 0.8812 to 0.8323 i.e., 4.89%. Similarly, as the repair rate (R_5_) increases from 0.002 to 0.014, the performability of the system increases simply from 0.8323 to 0.8561 i.e., 2.38%.

Validation of Results Obtained through Markov Method with the Help of ANN

In current research work, the ANN technique is utilized to check the adequacy of the final results.

It uses a two-layer feed-forward network for solving the data fitting problems. In addition, it also helps in dividing the data set into training and testing data sets. MATLAB software is utilized for employing the ANN technique on the given data set. The outcomes of the present study are mentioned below:

The predicated output performability levels obtained through ANN for various systems of RGTPP are compared with the output of performability levels through the Markov method. The error is attained by the subtraction of the performability levels (obtained through the Markov approach) and predicated output performability levels (obtained through ANN). The predicated output performability levels obtained through ANN are much closer to the output of performability levels obtained through the Markov method. The error between the performability levels obtained through the Markov method and ANN is very less. So, this small variation of performability levels validates the output of the Markov method with the help of ANN.

Table 7*(in appendix)* indicates the maximum error value i.e., - 4.97% for steam generation system. The corresponding values of FRR are as F_1_ = 0.011, F_2_ = 0.00022, F_3_ = 0.001, F_4_ = 0.008, F_5_ = 0.0003, R_1_ = 0.25, R_2_ = 0.002, R_3_ = 0.4, R_4_ = 0.11 andR_5_ = 0.008.

## Performability optimization

In the recent past, Particle Swarm Optimization has been found to be the most effective technique applied in many engineering and management applications for the optimization of the processes. This learning algorithm is based on the flying birds as the birds change their direction and control their speed as per their past experience to locate their destination by minimizing the distance gradually. The searching algorithm considers each solution as a particle (bird), and the fitness value of each particle has been estimated with the help of the fitness function. By tuning the cumulative speed and the position of the particle, the group performance (g-best) and the best performance of the individual (p-best) have been estimated. Multiple iterations have been performed to control the cumulative speed and the position of the particle. The speed and the position of *i_th_* particle for the population size of ‘*n’* are estimated by the following relations:

$$S_{i}=S_{i,1}+S_{i,2}+\_\_\_\_\_\_\_\_\_\_\_S_{i,n}$$

$$D_{i}=D_{i,1}+D_{i,2}+\_\_\_\_\_\_\_\_\_\_\_D_{i,n}$$

The particles are continuously allowed to move in the multidimensional search space, and with the help of successive iterations, the best optimum solution has been obtained.

In the present work, an attempt has been made to optimize the performability level for a thermal power plant i.e., by estimating the various groupings of FRR and multiple iterations on different population sizes. This problem of the thermal power plant has been considered 10-dimensional space of different failure rates and repair rates, as shown in Table 7. The constrained range of different FRR parameters to optimize the performability of the thermal power plant are described below:

**Table 7:** Range of various FRR parameters.

Parameters	F_1_	F_2_	F_3_	F_4_	F_5_	R_1_	R_2_	R_3_	R_4_	R_5_
Lower limit	0.007	0.00018	0.0008	0.006	0.0001	0.23	0.001	0.2	0.09	0.002
Upper Limit	0.015	0.00022	0.0013	0.010	0.0005	0.27	0.005	0.6	0.13	0.014

The position and the speed of the particles is reconfigured with the help of the following algorithms.

$$S_{i}=w\times S_{i}+c_{1}\times r_{1}\times p_{best\;i}-D_{i}+c_{2}\times r_{2}\times g_{best\;i}-D_{i}$$

$$D_{i}=S_{i}\times D_{i}$$

Where ‘*w*’ is the Inertia Weight, ‘*c_1_*_’_ is the Cognitive Parameter, ‘*c_2_*’ is the Social Parameter, and ‘*r_1_ & r_2_*’ are the Random Numbers arbitrarily selected.

In this part of the optimization technique, the algorithm has been terminated either at the maximum count of generations or at a minimum value of fitness function. The different parameters considered for the PSO algorithm are shown in Table 8.

**Table 8:** Various PSO algorithm parameters for steam generation systems.

Sr. No.	Parameter	Range/ Value	Remarks
1	Population Size	5-50	To find population size for optimum Performability
2	Number of Generations	10-100	To find generation size for optimum Performability
3	Inertia Weight (w)	0.7298	Its value lies between 0 and 1
4	Cognitive Factor (c_1_)	1.4962	Selected Arbitrarily and lies between 0 and 2
5	Social Factor (c_2_)	1.4962	Selected Arbitrarily and lies between 0 and 2
6	Random Number (R_1_)	0.9706	Selected Arbitrarily and lies between 0 and 1
7	Random Number (R_2_)	0.0318	Selected Arbitrarily and lies between 0 and 1

**Fig. 8:**
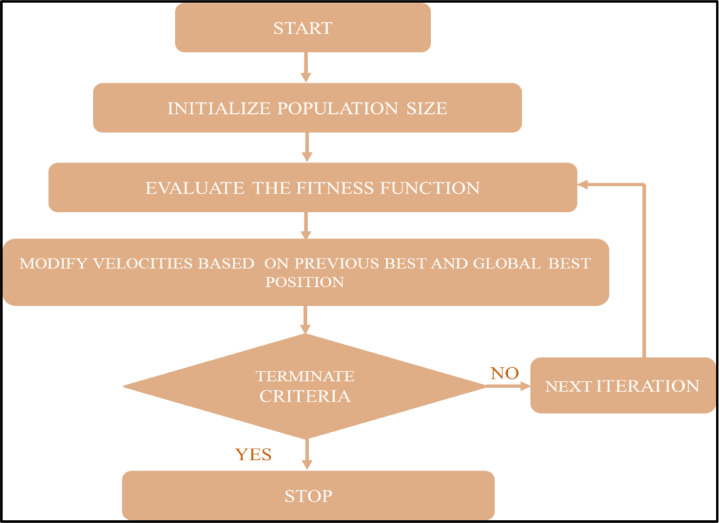
PSO Algorithm to optimize performability level for a thermal power plant.

The algorithm has been explained with the help of the following flow diagram as depicted in Figure 8.

By fine-tuning PSO parameters such as population size, step size of both the algorithms, and the number of iterations, the performability of the steam generation system has been optimized.

## Results and discussion

**Fig. 9:**
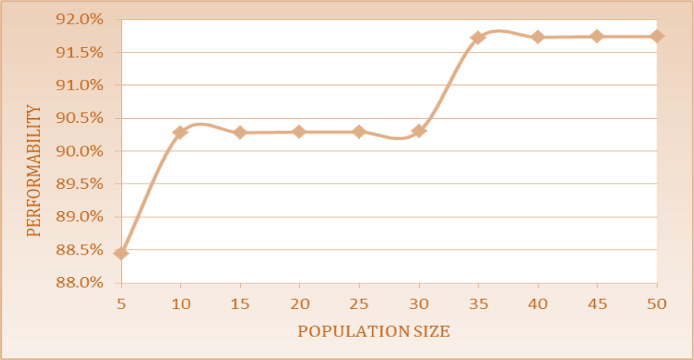
Impact of PS on system performability.

The optimum performability for steam generation system has observed 91.74% by using Particle swarm optimization approach at a PS of 45 and by taking GS constant i.e. 100. Table 9 indicates the best arrangements of FRR as F_1_ = 0.009, F_2_ = 0.0002, F_3_ = 0.0009, F_4_ = 0.0066, F_5_ = 0.0001, R_1_ = 0.3442, R_2_ = 0.0047, R_3_ = 0.5378, R_4_ = 0.0898 and R_5_ = 0.0085. Further, the performability of the system has been represented in Fig. 9 by taking the different parameters like PS and GS constant. The performability levels for steam generation system at PS varied from 5 to 50 in a step of 5 taking constant GS are specified as follows:

**Table 9:** Impact of constant GS (100) and PS on system performability.

PS	F_1_	F_2_	F_3_	F_4_	F_5_	R_1_	R_2_	R_3_	R_4_	R_5_	PA
5	0.0102	0.0002	0.0011	0.0077	0.0003	0.2262	0.0046	0.4362	0.1123	0.0097	88.44
10	0.009	0.0002	0.0008	0.0069	0.0002	0.3253	0.0038	0.339	0.1063	0.0076	90.28
15	0.0091	0.0002	0.0008	0.0069	0.0002	0.3259	0.0038	0.3359	0.1073	0.0076	90.28
20	0.009	0.0002	0.0008	0.0068	0.0002	0.3246	0.0038	0.3325	0.1073	0.0076	90.29
25	0.009	0.0002	0.0008	0.0068	0.0002	0.3232	0.0038	0.3318	0.1085	0.0076	90.29
30	0.0091	0.0002	0.0008	0.0068	0.0002	0.3251	0.0038	0.3298	0.1082	0.0076	90.3
35	0.009	0.0002	0.0009	0.0065	0.0001	0.3461	0.0047	0.528	0.0902	0.0085	91.72
40	0.009	0.0002	0.0009	0.0066	0.0001	0.3451	0.0047	0.5336	0.0896	0.0085	91.73
**45**	**0.009**	**0.0002**	**0.0009**	**0.0066**	**0.0001**	**0.3442**	**0.0047**	**0.5378**	**0.0898**	**0.0085**	**91.74**
50	0.009	0.0002	0.0009	0.0066	0.0001	0.3439	0.0047	0.5384	0.0898	0.0085	91.74

**Fig. 10:**
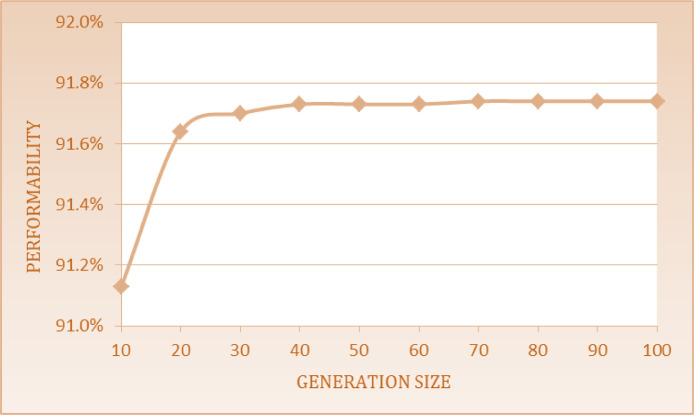
Impact of GS on system performability.

The optimum performability of steam generation system obtained is 91.74% by using Particle Swarm Optimization approach at GS (70) and PS constant i.e. 40. Table 10 shows the system performability variations with the best arrangements of FRR as F_1_ = 0.009, F_2_ = 0.0001, F_3_ = 0.0009, F_4_ = 0.0065, F_5_ = 0.0001, R_1_ = 0.345, R_2_ = 0.005, R_3_ = 0.534, R_4_ = 0.0895 and R_5_ = 0.0085. The impact of common parameters like PS and GS constant is shown in Fig. 10. The performability levels for steam generation system at GS varied from 10 to 100 in a step of 10 taking constant PS are given as follows:

**Table 10:** System performability variations with the best arrangements of FRR.

GS	F_1_	F_2_	F_3_	F_4_	F_5_	R_1_	R_2_	R_3_	R_4_	R_5_	PA
10	0.009	0.0002	0.0009	0.01	0.0001	0.3313	0.0045	0.5271	0.0909	0.0084	91.13
20	0.009	0.0001	0.0009	0.0065	0.0001	0.3399	0.0001	0.54	0.09	0.01	91.64
30	0.009	0.0001	0.0009	0.0065	0.0001	0.3436	0.005	0.537	0.089	0.009	91.7
40	0.009	0.0001	0.0009	0.0065	0.0001	0.34	0.001	0.53	0.09	0.0085	91.73
50	0.009	0.0001	0.0009	0.0065	0.0001	0.345	0.005	0.534	0.09	0.0085	91.73
60	0.009	0.0001	0.0009	0.0065	0.0001	0.35	0.001	0.53	0.0895	0.0085	91.73
70	**0.009**	**0.0001**	**0.0009**	**0.0065**	**0.0001**	**0.345**	**0.005**	**0.534**	**0.0895**	**0.0085**	**91.74**
80	0.009	0.0001	0.0009	0.0065	0.0001	0.3451	0.0047	0.5336	0.0895	0.0085	91.74
90	0.009	0.0001	0.0009	0.0065	0.0001	0.3451	0.0047	0.5336	0.0895	0.0085	91.74
100	0.009	0.0001	0.0009	0.0065	0.0001	0.3451	0.0047	0.5336	0.0895	0.0085	91.74

## Conclusions

In current research work, the Markov Birth death technique is utilized to find out the best economical maintenance schedule for thermal power plant steam generation system. The following conclusion is drawn listed below:

1. The most critical unit is found to be the economizer unit in which the availability increased from 0.7640 to 0.8827, i.e., (11.87 %), whereas boiler drum is observed as the least critical unit with an availability enhanced from 0.8627 to 0.8657 (i.e., 0.3 %).
2. A drastic fall of 2.41 % in the level of unit availability occurs with the rise in the failure rate of the economizer (F_2_) from 0.00018 to 0.00022. Also, a significant boost of 11.87% in unit availability can be observed with the rise in repair rate economizer (R_2_) from 0.001 to 0.005.
3. A fall of 1.9 % in the level of highe pressure heater availability occurs with the rise in the failure rate (F_1_) from 0.007 to 0.015. Also, a noticeable increase of 3.6% in unit availability can be observed with the rise in repair rate (R_1_) from 0.232 to 0.27.
4. A radical fall of 4.89 % in the level of unit availability occurs with the rise in failure rate of super heater subsystem (F_5_) from 0.0001 to 0.0005. Also, an appreciable enhancement of 2.38 % in unit availability can be observed with the rise of repair rate of super heater subsystem (R_5_) from 0.002 to 0.014.
5. A fall of 0.82% in the level of unit availability occurs with the rise in failure rate of centrifuge subsystem (F_4_) from 0.006 to 0.010. Also, a substantial improvement of 1.01 % in unit availability can be observed with the rise of repair rate of centrifuge (R_4_) from 0.09 to 0.13.
6. A marginal fall of .15% in the level of unit availability occurs with the rise in failure rate of sugar grader (F_3_) from 0.0008 to 0.0012. Also, a mere step up of 0.03 % can be observed in unit availability with the rise in repair rate of sugar grader (R_3_) from 0.2 to 0.6.
7. For the complete analysis of thermal power plant, it can be arranging the different units of thermal power plant according to maintenance priorities as follows: economizer, high pressure heater, super heater, boiler tubes, and boiler drum.
8. The difference/error between the results obtained through the Markov method and ANN is very less, up to 5%. So, this small variation validates the output of the Markov method with the help of ANN. Furthermore, the performability of the concerned system has been optimized by using the PSO algorithm to improve the total performance of the concerned system. It is observed that the highest performability level, i.e., 91.74 at a population size of 40 and at a generation size of 70.

## Declaration of Competing Interest

There is no conflict of interest on this article.

## Figures and Tables

**Table 11:** Comparison of performability levels obtained through markov method and ANN for steam generation system.

S. No.	R_1_	R2	R3	R4	R5	F_1_	F2	F3	F4	F5	Markov Method	ANN	Error
	0.25	0.003	0.4	0.11	0.008	0.011	0.0002	0.001	0.008	0.0003			
1	0.23	0.003	0.4	0.11	0.008	0.007	0.0002	0.001	0.008	0.0003	0.8535	0.8666	-0.0131
2	0.24	0.003	0.4	0.11	0.008	0.007	0.0002	0.001	0.008	0.0003	0.8646	0.8708	-0.0062
3	0.25	0.003	0.4	0.11	0.008	0.007	0.0002	0.001	0.008	0.0003	0.8714	0.8727	-0.0013
4	0.26	0.003	0.4	0.11	0.008	0.0077	0.0002	0.001	0.008	0.0003	0.8760	0.8724	0.0036
5	0.27	0.003	0.4	0.11	0.008	0.007	0.0002	0.001	0.008	0.0003	0.8793	0.8704	0.0089
6	0.23	0.003	0.4	0.11	0.008	0.009	0.0002	0.001	0.008	0.0003	0.8487	0.8675	-0.0188
7	0.24	0.003	0.4	0.11	0.008	0.009	0.0002	0.001	0.008	0.0003	0.8609	0.8690	-0.0081
8	0.25	0.003	0.4	0.11	0.008	0.009	0.0002	0.001	0.008	0.0003	0.8684	0.8691	-0.0007
9	0.26	0.003	0.4	0.11	0.008	0.009	0.0002	0.001	0.008	0.0003	0.8734	0.8675	0.0059
10	0.27	0.003	0.4	0.11	0.008	0.009	0.0002	0.001	0.008	0.0003	0.8771	0.8646	0.0125
							–						
							–						
							–						
40	0.25	0.005	0.4	0.11	0.008	0.011	0.0002	0.001	0.008	0.0003	0.8858	0.8674	0.0184
41	0.25	0.001	0.4	0.11	0.008	0.011	0.00021	0.001	0.008	0.0003	0.7699	0.8164	-0.0465
42	0.25	0.002	0.4	0.11	0.008	0.011	0.00021	0.001	0.008	0.0003	0.8376	0.8720	-0.0344
43	0.25	0.003	0.4	0.11	0.008	0.011	0.00021	0.001	0.008	0.0003	0.8629	0.8737	-0.0108
44	0.25	0.004	0.4	0.11	0.008	0.011	0.00021	0.001	0.008	0.0003	0.8761	0.8740	0.0021
45	0.25	0.005	0.4	0.11	0.008	0.011	0.00021	0.001	0.008	0.0003	0.8842	0.8738	0.0104
46	0.25	0.001	0.4	0.11	0.008	0.011	0.00022	0.001	0.008	0.0003	0.7640	0.8123	-0.0483
**47**	**0.25**	**0.002**	**0.4**	**0.11**	**0.008**	**0.011**	**.00022222**	**0.001**	**0.008**	**0.0003**	**0.8341**	**0.8838**	**-0.0497**
48	0.25	0.003	0.4	0.11	0.008	0.011	0.00022	0.001	0.008	0.0003	0.8604	0.8838	-0.0234
49	0.25	0.004	0.4	0.11	0.008	0.011	0.00022	0.001	0.008	0.0003	0.8742	0.8837	-0.0095
50	0.25	0.005	0.4	0.11	0.008	0.011	0.00022	0.001	0.008	0.0003	0.8827	0.8839	-0.0012
							–						
							–						
							–						
115	0.25	0.003	0.4	0.11	0.014	0.011	0.0002	0.001	0.008	0.0003	0.8710	0.8644	0.0066
116	0.25	0.003	0.4	0.11	0.002	0.011	0.0002	0.001	0.008	0.0004	0.8440	0.8541	-0.0101
117	0.25	0.003	0.4	0.11	0.005	0.011	0.0002	0.001	0.008	0.0004	0.8509	0.8616	-0.0107
118	0.25	0.003	0.4	0.11	0.008	0.011	0.0002	0.001	0.008	0.0004	0.8561	0.8613	-0.0052
119	0.25	0.003	0.4	0.11	0.011	0.011	0.0002	0.001	0.008	0.0004	0.8602	0.8577	0.0025
120	0.25	0.003	0.4	0.11	0.014	0.011	0.0002	0.001	0.008	0.0004	0.8635	0.8559	0.0076
121	0.25	0.003	0.4	0.11	0.002	0.011	0.0002	0.001	0.008	0.0005	0.8323	0.8544	-0.0221
122	0.25	0.003	0.4	0.11	0.005	0.011	0.0002	0.001	0.008	0.0005	0.8407	0.8595	-0.0188
123	0.25	0.003	0.4	0.11	0.008	0.011	0.0002	0.001	0.008	0.0005	0.8470	0.8575	-0.0105
124	0.25	0.003	0.4	0.11	0.011	0.011	0.0002	0.001	0.008	0.0005	0.8520	0.8531	-0.0011
125	0.25	0.003	0.4	0.11	0.014	0.011	0.0002	0.001	0.008	0.0005	0.8561	0.8508	0.0053

## Data Availability

No data was used for the research described in the article.
